# Concept for quantifying the dose from image guided radiotherapy

**DOI:** 10.1186/s13014-015-0492-7

**Published:** 2015-09-17

**Authors:** Uwe Schneider, Roger Hälg, Jürgen Besserer

**Affiliations:** Institute of Physics, Science Faculty, University of Zürich, Zürich, Switzerland; Radiotherapy Hirslanden, Witellikerstrasse 40, CH-8032 Zürich, Switzerland

## Abstract

**Background:**

Radiographic image guidance is routinely used for patient positioning in radiotherapy. All radiographic guidance techniques can give a significant radiation dose to the patient. The dose from diagnostic imaging is usually managed by using effective dose minimization. In contrast, image-guided radiotherapy adds the imaging dose to an already high level of therapeutic radiation which cannot be easily managed using effective dose. The purpose of this work is the development of a concept of IGRT dose quantification which allows a comparison of imaging dose with commonly accepted variations of therapeutic dose.

**Methods:**

It is assumed that dose variations of the treatment beam which are accepted in the spirit of the ALARA convention can also be applied to the additional imaging dose. Therefore we propose three dose categories: Category I: The imaging dose is lower than a 2 % variation of the therapy dose. Category II: The imaging dose is larger than in category I, but lower than the therapy dose variations between different treatment techniques. Category III: The imaging dose is larger than in Category II. For various treatment techniques dose measurements are used to define the dose categories. The imaging devices were categorized according to the measured dose.

**Results:**

Planar kV-kV imaging is a category I imaging procedure. kV-MV imaging is located at the edge between category I and II and is for increasing fraction size safely a category I imaging technique. MV-MV imaging is for all imaging technologies a category II procedure. MV fan beam CT for localization is a category I technology. Low dose protocols for kV CBCT are located between category I and II and are for increasing fraction size a category I imaging technique. All other investigated Pelvis-CBCT protocols are category II procedures. Fan beam CT scout views are category I technology. Live imaging modalities are category III for conventional fractionation, but category II for stereotactic treatments.

**Conclusions:**

Dose from radiotherapy imaging can be categorized in terms of generally accepted dose variations of therapy dose. This concept allows the quantification of daily dose from image guided radiotherapy in the spirit of the ALARA convention.

## Background

In radiation therapy the integral dose is increasing steadily with the introduction of more imaging procedures to the treatment process [1]. Image-guided radiotherapy (IGRT) makes use of many different imaging techniques, using modalities as portal imaging, fluoroscopy and CT in many variations (fan-beam, cone-beam, MV, kV). Clinically applied regimens are as simple as a single setup image or as complex as intrafraction tumor tracking. The total imaging radiation dose experienced by a patient can include multiple CT scans for planning, pretreatment fluoroscopic studies to analyze tumor motion, and a series of interfraction and intrafraction images for target localization.

The quantification of the additional imaging dose in terms of second cancer risk for the patient is not an easy task. The report of the AAPM task group 75 [1] on the management of imaging dose during image-guided radiotherapy recommends to use the concept of effective dose to determine the stochastic risk for the patient. It is fundamentally important to compare the imaging dose to the total dose delivered by the treatment beams themself. The AAPM task group 75 [1] discourages from naively equating imaging dose with treatment beam, scatter, and leakage dose, since the comparison process is more complicated. As the dose-response curve for radiation induced cancer might be different at therapeutic dose levels when compared to imaging doses, it is also not recommended to use effective dose to quantify therapy dose in order to compare it to the effective dose from imaging. Therefore the task group 75 [1] considers that it is not yet practical to quantitatively combine the evaluation of total imaging dose with the evaluation of total therapeutic dose. However, to manage imaging dose in the spirit of the ALARA convention it is necessary to establish a quantitative comparison between imaging and therapeutic dose.

The aim of this report is the development of a concept which allows a comparison of imaging dose with commonly accepted variations of the treatment beam, scatter, and leakage dose. It is assumed that the dose variations of the treatment beam which are accepted by the radiation oncologist and/or medical physicist in the spirit of the ALARA convention can also be applied to the additional imaging dose.

## Methods

Various measurements of peripheral imaging and treatment dose have been published recently [2–6]. In this report we focus for analysis on the measurements of Hälg et al. [6] for the following reasons. Hälg et al. measured a three-dimensional dose distribution in the Alderson phantom with 183 TLDs. The radiotherapy treatment plans which were irradiated were planned for an adolescent patient with a Rabdomyosarcoma in the prostate. Thus the irradiated PTV was relatively small and as a consequence the ratio of imaging to therapy dose large (the impact of imaging is conservatively estimated). In addition not only various radiotherapy treatment techniques, but also the dose from treatment machines of several vendors was measured. The measured imaging modalities, listed in Table 1, included planar, CT and fluoroscopic imaging with kV and MV beams, respectively. Details of the measurements can be found in the publications of Hälg et al. [6, 7].

**Table 1:** Analysed imaging devices and corresponding imaging techniques used in this study. The machine parameters and settings for each imaging modality can be found in [6]

Imaging device	Imaging technique
Accuray cyberknife	Stereoscopic kV planar images
Accuray tomotherapy	MV fan beam CT
Elekta synergy	MV portal images
Elekta XVI	kV CBCT
GE HiSpeed DX/i	CT scout views
Siemens oncor avant-garde	MV CBCT and portal images
Varian clinac 21 iX	MV portal images
Varian OBI	kV CBCT and planar images
Varian truebeam	kV CBCT

The distribution of dose in the patient depends on several factors. In planar imaging the dose to the patient is greatest at the skin surface nearest to the source and falls off progressively as the radiation transits the body to the image detector. Axial imaging, which is the basis for CT, differs in that the dose, by design, is distributed nearly uniformly throughout the imaged volume to produce 3D images of uniform cross-sectional quality. Therefore dose in planar imaging is concentrated at the skin while dose for tomography is distributed more in the manner of 3D radiation therapy [1]. A remaining question is therefore how three-dimensional imaging and therapy dose distributions can be evaluated. For this reason the three-dimensional measurements were averaged in cross-sectional slices with increasing distance from the irradiated volume (PTV) in 2.5 cm steps (form feet to head). The averaged dose was then compared to the measured on-axis-dose in the superior-inferior direction reaching from the edge of the PTV to the head in 2.5 cm steps.

For the quantification of imaging dose in radiotherapy we propose three categories:

1. Category I: The imaging dose is lower than a 2 % variation of the therapy dose.
2. Category II: The imaging dose is larger as defined by category I, but lower than the variation of therapy dose between different treatment techniques.
3. Category III: The imaging dose is larger than the variation of therapy dose between different treatment techniques which are clinically in use.

The reason for the definition of category I is the internationally accepted quality standard for radiotherapy beam output variations of medical electron accelerators. Many national protocols use 2 % as an upper limit for output variations, which is, among others, defined based on the ALARA principle [8–11]. Thus it is commonly accepted that radiotherapy scatter and leakage can be 2 % higher than originally planned. We argue that an additional imaging dose which is lower than the well accepted 2 % therapy dose variation does not need additional justification.

In current clinical practice the choice of treatment technique determines the amount of peripheral dose to the patient. For example an IMRT treatment plan delivers more scatter and leakage dose than a conformal 3D plan. This additional dose is usually justified by a better sparing of the organs at risk.

The ALARA principle is usually of low priority in the choice of a treatment machine. However, the dose variation originating from different treatment machines while using the same treatment technique is even larger than the variation with treatment technique (as will be seen later). Therefore category II includes imaging modalities which deliver a dose to the patient which is below the dose variation originating from different treatment techniques. This can provide a tool for justifying the additional imaging dose if the benefit for the patient is similar to the benefit from a modern treatment technique.

The treatment techniques, treatment machines and imaging technologies for which the three-dimensional dose distribution was measured are listed in Table 1.

The magnitude of the therapeutic dose distribution is proportional to the dose per fraction and thus the ratio of imaging to therapeutic dose will depend on fraction size. Therefore the categorization of imaging dose was performed for 2, 5 and 10 Gy per fraction, respectively.

## Results

**Fig. 1:**
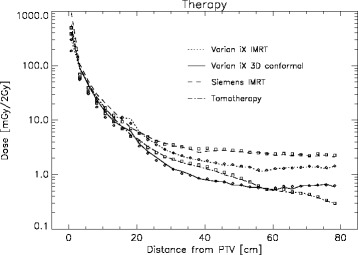
Central axis therapy dose is plotted for a selection of treatment techniques and manufacturers as a function of the distance from the PTV as the solid lines for a 2 Gy fraction size. The symbols represent the average dose in the corresponding cross-sectional slice in the phantom

In Fig. 1 the lines represent the measured central axis therapy dose in mGy for a 2 Gy therapy fraction for a selection of treatment techniques and machine manufacturers. The dose is plotted as a function of the distance from the PTV. The corresponding symbols show the average dose in the corresponding cross-sectional slice in the phantom. Two things can be recognized. First, the dose variation between different radiotherapy techniques (Varian 3D conformal treatment and Varian IMRT) and between different treatment machines using the same technique (IMRT with Varian, Siemens and Tomotherapy) are significant [7]. Second the central axis dose is in satisfying agreement with the average of the three-dimensional dose distribution in each slice. Therefore we believe that the central axis dose is a good measure of the deposited energy per area from a therapeutic irradiation of a co-planar treatment.

**Fig. 2:**
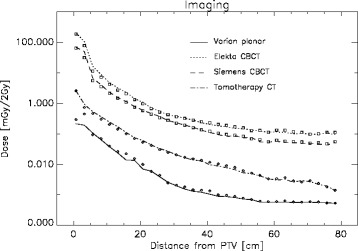
Central axis imaging dose is plotted for a selection of imaging modalities and manufacturers as a function of the distance from the PTV as the solid lines for a 2 Gy fraction size. The symbols represent the average dose in the corresponding cross-sectional slice in the phantom

The same is shown in Fig. 2 for several imaging modalities. The conclusions from therapy can be applied also to imaging. The central axis depth dose curve is representative for the total deposited energy per slice and large variations between the different imaging modalities can be observed.

**Fig. 3:**
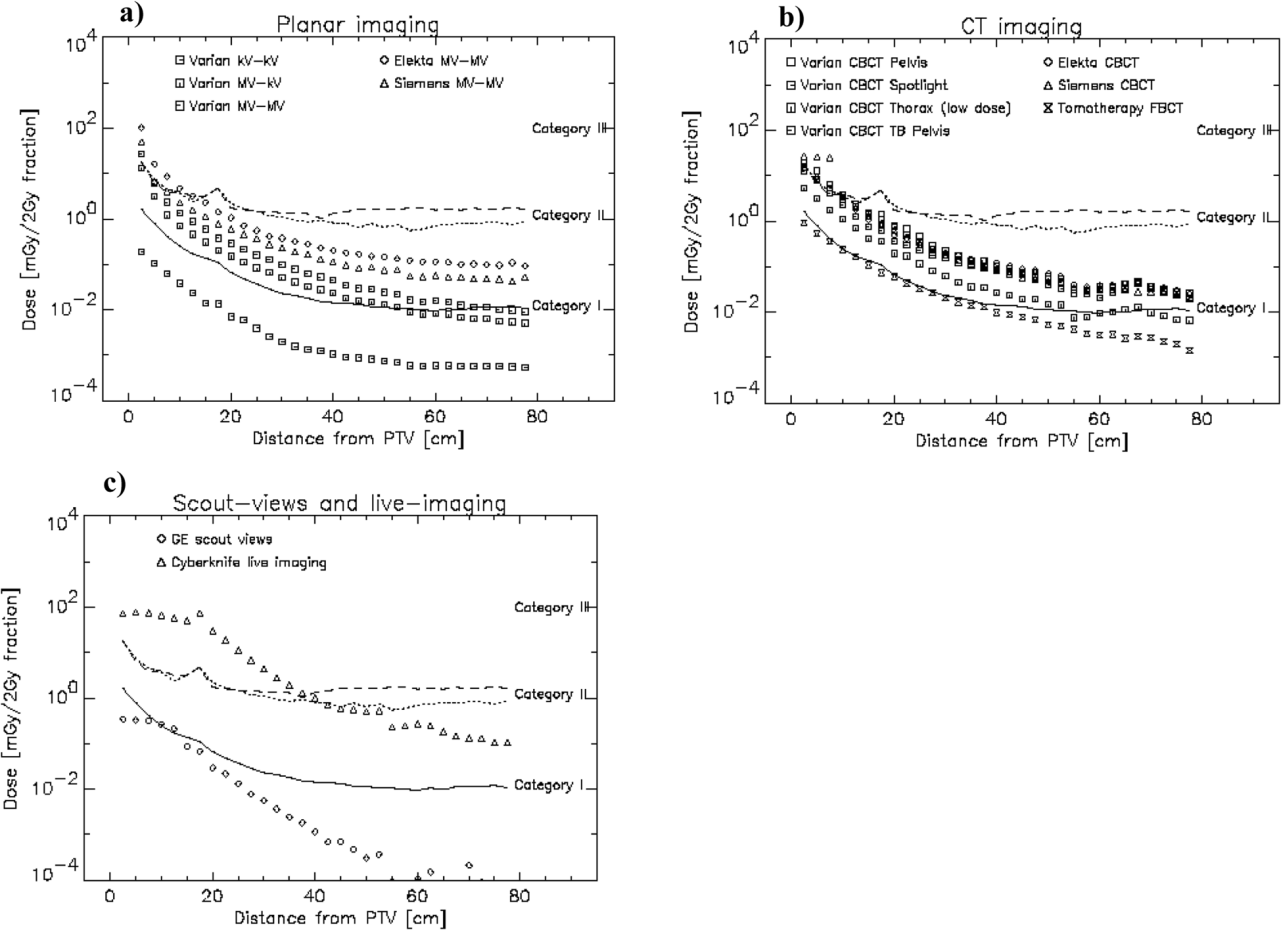
Symbols represent the imaging dose as a function of the distance from the PTV for a 2 Gy fraction. The lines denote the borders between the different dose categories. The category I/II and the category II/III-transition are represented by the solid and the dashed/dotted lines, respectively. The solid line represents the 2 % dose variation of linac output, the dotted line the variation of treatment techniques and the dashed line the variation between machine types. Planar imaging modalities are depicted in **a**), CT-imaging in **b**) and scout view and live imaging in **c**)

In a next step the 2 % variation of the therapy dose from a 3D conformal Varian treatment was calculated from the measurements. The 3D conformal plan was chosen, as it corresponds to the lowest scatter and leakage dose when compared to the other treatment options. Thus it is a conservative estimate of the 2 % limit and any other technique, as VMAT or IMRT would result in a larger 2 % dose contribution. In Fig. 3 this 2 % dose which defines the upper limit for category I is plotted as the solid line as a function of the distance from the irradiated target volume on the central axis of the Alderson phantom. For the category II limit the maximum dose difference between Varian iX 3D-CRT, Varian iX IMRT, Varian IX VMAT, Varian Truebeam FFF VMAT) was determined and plotted as the dotted line. In addition the dose difference between machines of different vendors (Varian, Elekta, Siemens and Accuray Tomotherapy) for the IMRT treatment technique is plotted as the dashed line in the Fig. 3.

In Fig. 3a the dose contribution of planar imaging is shown for kV and MV energies and different vendors for a 2 Gy fraction size. In Fig. 3b different CT imaging modalities are plotted including cone beam and fan beam CT, low-and high dose CBCT and also the CT dose from machines of different vendors. Finally Fig. 3c shows for 2 Gy fraction size the dose of a CT scout view which is sometimes used in proton therapy for positioning purposes and the dose from Cyberknife live imaging.

**Fig. 4:**
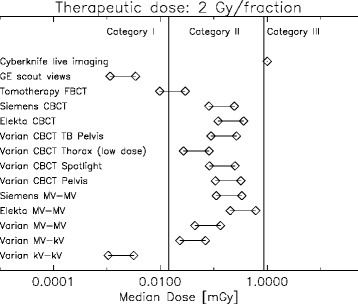
Classification of various imaging modalities according to median dose in dose categories for a therapeutic dose of 2 Gy per fraction. Two symbols per imaging modality are plotted representing the increase of dose when imaging is applied three times per fraction instead of one

For a better comparison of the imaging modalities the dose variation along the central axis was averaged. As the additional cancer risk from imaging is supposed to be a function of dose and volume the dose averaging was performed in terms of median dose. The resulting median doses are plotted in Fig. 4 for a 2 Gy fraction. For each imaging modality or combination of images two symbols were plotted representing the dose for one or three applications per fraction, respectively.

**Fig. 5:**
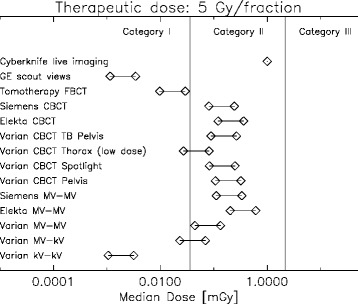
Classification of various imaging modalities according to median dose in dose categories for a therapeutic dose of 2 Gy per fraction. Two symbols per imaging modality are plotted representing the increase of dose when imaging is applied three times per fraction instead of one

**Fig. 6:**
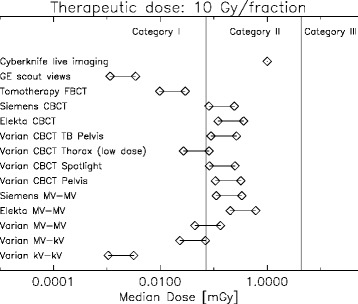
Classification of various imaging modalities according to median dose in dose categories for a therapeutic dose of 10 Gy per fraction. Two symbols per imaging modality are plotted representing the increase of dose when imaging is applied three times per fraction instead of one

The impact of daily imaging is decreasing with increasing fraction size. Therefore the boundaries of the dose categories are shifted to larger doses when moving from conventional to hypofractionation. This can be seen in Figs. 5 and 6 where the median imaging doses are shown for a 5 Gy and a 10 Gy fraction, respectively.

## Discussion

The classification of the different imaging techniques for image guided radiotherapy is shown in Figs. 4, 5, and 6. It can be concluded that planar kV-kV imaging is independent of fraction size always a category I imaging procedure. kV-MV imaging is located at the edge between category I and II and is for increasing fraction size safely a category I imaging technique. MV-MV imaging, however, is for all imaging technologies a category II procedure.

The dose optimized Tomotherapy MV fan beam CT for localization is low in dose and a category I technology. Low dose protocols for kV CBCT are located between category I and II and its classification may vary according to fractionation. All other investigated Pelvis-CBCT protocols are category II procedures.

Fan beam CT scout views which are sometimes used for patient setup in proton therapy are category I. However, live imaging modalities must be viewed with care, even when the fraction number is small and large dose fractions are used. Although live imaging with 400 single images is for a 10 Gy fractionation schedule in category II, it should be noted that the dose up to around 20 cm distance from the treated volume is more or less constant (Fig. 3c) which is not reflected in the median dose.

This study has limitations that warrant consideration. The dose measurements which were used in this study were performed in an Alderson phantom for one indication. The therapeutic scatter and imaging dose varies with PTV volume, PTV localisation and size of the patient. Therefore the IGRT dose presented in this study cannot represent all clinical circumstances. In this work a target in an adolescent patient with a relatively small PTV was evaluated. The analysed imaging dose was therefore large relative to the therapeutic dose and represents a conservative dose estimate.

It is important to note that radiation sensitivity varies strongly with patient age. Children and adolescent patients are several times more sensitive than adults. Therefore the dose categories presented in this work should be used as an aid to apply the ALARA concept, the imaging dose, however, should be managed on a patient-by-patient basis.

In this work it was assumed that the size of the PTV is independent of the used set-up procedure. However, the application of different levels of image guidance allows the reduction of the safety margins which define the size of the PTV. As a consequence the integral dose of a radiotherapy treatment using image guidance can be smaller than that without image guidance. This may somehow compensate for the additional imaging dose. Unfortunately such effects cannot be quantified until we have a new concept for combining therapy and imaging dose into a risk equivalent quantity.

## Conclusions

In this work a concept was developed to quantify daily dose from image guided radiotherapy in the spirit of the ALARA convention. Three dose categories are proposed: category I which is defined by a dose contribution smaller than a 2 % variation of the therapeutic dose, category II which is defined by the dose variation between different treatment techniques and category III which exceeds the dose level of category II. It is proposed that IGRT dose contributions which fall into category I do not need additional justification. If the imaging dose is in category II the additional imaging dose is justified if the benefit for the patient is similar to the benefit from the application of a modern treatment technique. Imaging procedures which fall into category III need additional justification.

It can be concluded that none of the investigated imaging procedures, except the live imaging, falls into category III. Most imaging methods, including the Pelvis CBCT protocols and MV-MV planar imaging are category II and will add a similar amount of extra dose to the patient than an IMRT treatment when compared to 3D conformal radiotherapy. Optimized low dose CBCT, MV fan beam CT (of Tomotherapy) and kV-kV planar imaging are category I modalities which, in our opinion, do not need additional justification.
